# How do Lebanese patients perceive the ideal doctor based on the CanMEDS competency framework?

**DOI:** 10.1186/s12909-019-1837-y

**Published:** 2019-10-29

**Authors:** Mabel Aoun, Ghassan Sleilaty, Simon Abou Jaoude, Dania Chelala, Ronald Moussa

**Affiliations:** School of Medicine, Saint-Joseph University, Beirut, Lebanon

**Keywords:** CanMEDS, Medical education, Patient perception, Competencies, Eastern Mediterranean population, Professionalism, Communication, Ethics

## Abstract

**Background:**

During their training, Lebanese medical students develop a high medical expertise but are not focusing on other competencies such as communication, collaboration, erudition, professionalism, leadership and health promotion. There is also insufficient data about patients’ preference for these skills. This study describes the different weights patients attribute to these physician’s competencies.

**Methods:**

This is a cross-sectional study based on a questionnaire distributed to 133 Lebanese patients. It included 15 questions assessing how patients prioritize the physician’s competencies, with open-ended questions asking them to define “the good doctor”. Krippendorff’s alpha coefficient was used to analyze the reliability of the competencies’ classification.

**Results:**

One hundred twenty five patients completed the questionnaire in this cross-sectional study. Their mean age was 48 ± 16.76 years. When classifying competencies, 73.6% opted for medical expertise as first choice and 48% put communication as second. Based on the Krippendorff’s coefficient, we identified a moderate agreement for the seven choices (alpha = 0.44). In open-ended questions, patients defined the good doctor in 325 answers: 64.3% mentioned medical expertise, 34.1% high ethics and 26.2% communication.

**Conclusions:**

This patient-centered study concurs well with the worldwide practice that puts medical expertise at the center of medical education. However Lebanese patients don’t perceive equally other competencies and favor professionalism and communication that should be integrated in priority in students’ curricula.

## Background

Medical education has largely evolved in the last decades and is focusing more on a training that takes outcomes into consideration. These ultimately attained outcomes are competencies or abilities that a successful physician should possess in order to enhance patient care. The core of these competencies is the clinical one known as medical expertise that requires a vast amount of knowledge and clinical skills brought to medical students during their long years of training. Medical expertise alone is insufficient and needs to be backed up with non-clinical competencies that will lead a doctor’s performance to an excellence stage. These key non-clinical qualities have been defined by the Canadian Medical Education Directions for Specialists (CanMEDS) physician competency framework and include the roles of communicator, collaborator, leader, scholar, professional and health advocate [1–10].

The CanMEDs framework has been developed by The Royal College of Physicians and Surgeons in Canada in 2005, updated in 2015 and has been embraced not only by Canada’s schools of medicine but also by several European, Australian, Asian and Eastern Mediterranean faculties [11–14]. Its ultimate goal is to improve the standards of medical practice by following a competency-based medical education model. In the United States, since 1999, another comprehensive competency framework is used in the training and assessment of students called the Accreditation Council for Graduate Medical Education (ACGME) [15]. In the Netherlands, the schools of medicine are following the competency framework for undergraduate medical education and those in the United Kingdom the general medical council [16, 17]. In Lebanon, the Saint-Joseph University of Beirut started recently an initiative to develop a competency-based medical education program that responds best to the needs of the Lebanese population. This new educational approach plans for a gradual integration of non-clinical competencies into the curriculum of medical students.

Competencies that should be introduced in priority will depend on the international evidence, the medical teachers’ assessment of the context, the student’s opinion and eventually the patient’s needs and perceptions. In fact, the “ideal doctor” can be perceived differently by a medical student, a nurse, a practicing physician or an academic healthcare provider [18–23]. Students in the Netherlands for instance prioritized the communication and professionalism roles whereas academic physicians picked up the communication as the most important [19, 20]. So far, patients have never been asked to put in order of priority the 7 competencies of the CanMEDS framework.

Taking into account the patient’s perception may allow the Lebanese schools of medicine to improve the curricula of their medical students based on contextualized evidence. The aim of this study is to find out how Lebanese patients perceive the good doctor and what are the competencies they would prefer to see as a priority in their treating physician.

## Methods

### Study design, setting and participants

This is a cross-sectional study that took place in 14 Lebanese clinics and included in each clinic the first 10 outpatients more than 18 years old who were visiting a doctor at the clinics in July 2018 and who gave their consent to fill the questionnaire. Lebanon is a Middle-Eastern country with 4.5 million inhabitants based on World Bank 2017. The Lebanese health system is mainly a private one with few services in private hospitals reimbursed by the government. The private clinics were located in hospitals of rural and urban areas covering the majority of the Lebanese territory: Beirut, Mount-Lebanon, South, North and Beqaa. Those clinics were chosen for being convenient to the researchers who have direct contact with the corresponding physicians thus facilitating their consent to participate. The specialties of physicians were: two nephrologists, one dermatologist, four general practitioners, one gastro-enterologist, one orthopedist, one endocrinologist, one cardiologist, one gynecologist, one internal medicine and one general surgeon. After the physician’s consent, the patients needed to give their consent as well. Questionnaires were given to patients by the secretaries at the clinics. Patients were excluded if not mentally capable of understanding the questions (neurological disorder) based on the statement of a family member. Patients had the choice to take the questionnaire home and give it back later.

### Data collection and questionnaire

Data were collected anonymously from patients who walked in the clinics. The questionnaire included 13 closed- and 2 open-ended questions (Additional file 1 and Additional file 2). It was written in simple Arabic/Lebanese and the 7 competencies were highlighted in Arabic and French (because French is the second language in the country).

A pilot study on 30 Lebanese adults (non-physicians) was performed. The 30 individuals filled the questionnaire and put their comments to improve it. Many respondents suggested to add “neutral” to two questions on preferences and many were confused with the order of questions so we adjusted it according to their suggestions.

The first ten questions aimed to assess the demographics and education of the patients, the demographics and specialty of their main treating physician and their preference for age and gender of an eventual future physician.

In questions 11 and 12 the patients were asked to put in order of priority, based on their own perception, the seven CanMEDS competencies. Since Lebanese medical schools have not yet established a competency framework, this study used the CanMEDS framework among others that are being assessed at the national level. The CanMEDS updated in 2015 included seven competencies: the medical expertise at the center, supported by the roles of communicator, collaborator, leader, scholar, professional and health advocate [1]. Patients were asked also to prioritize the components or sub-elements of each competency based on their description in the CanMEDS 2015. The different components of each competency are summarized in the questionnaire (Additional file 1 and Additional file 2).

Question 13 asked the patients to give a score to the importance of the physician’s smile, on a scale from 0 to 10.

Questions 14 and 15 were open-ended: “What are the qualifications that make you say the doctor is competent?” “What are the qualifications that make you say the doctor is not competent?”

### Sample size

Since the adult Lebanese population includes ~ 3 million people, if we consider a confidence interval of 0.06 and a confidence level of 90% and we assume that 80% of the adult population have encountered a physician at one point in time [24], the sample size needed to be representative of the population would be 121. Taking into consideration a 10% of non-responders, the final study size would need at least 133 patients. In order to get equal number of 10 patients in each of the 14 specialists’ clinics, we included a total of 140 patients.

### Ethical considerations

This study got the approval of the Saint- Joseph University ethics committee (CEHDF 1194). It is in agreement with the declaration of Helsinki. All patients gave their consent before participation and data were collected anonymously.

### Statistical analysis

The Statistical Package for Social Sciences (SPSS) version 24.0 was used for data entry and statistical analysis. Continuous variables were expressed as means and standard deviations (SD) if normally distributed and as median and interquartile range (IQR) if data was skewed. Categorical variables were summarized as numbers and percentages. The Krippendorff’s alpha coefficient was used to test the reliability of patients’ choices when classifying the seven competencies (with 1 being highly reliable and 0.000 non-reliable) [25]. Chi-square test was used to compare the difference between female and male patients in their choice of competencies. T independent test was used to compare the age between different groups. *P* < 0.05 was considered as statistically significant. As for the data collection of the open-ended questions, every new theme was entered as a new variable and themes were then bundled into the 7 competencies’ domains.

## Results

### Socio-demographic characteristics of patients

One hundred forty forms were distributed and 125 patients completed the questionnaire. 68.8% lived in the Mount-Lebanon area, the governorate that includes the highest number of inhabitants. Table 1 summarizes their general characteristics. Their mean age was 48 ± 16.76 years, varying between 19 and 85 years. 56% were female and 44% were male. 68.8% went to college and 61.6% worked. Regarding their treating physicians, 74.4% had a physician of male gender, 68.8% were indifferent regarding the gender of their physician and the majority of them (70.4%) had a preference for a 40- to 60-year-old doctor. 61.6% consulted their physician more than once a year.

**Table 1:** General characteristics of the 125 patients

	Total *n* = 125
Age (years),	
*Mean ± SD*	48 ± 17
*Median (IQR)*	46 (35, 60)
Sex (M/F)	55 / 70
College (%)	68.8%
Work (%)	61.6%
Treating physician’s sex (M/F)	93 / 32
Age of treating physician,	
Mean ± SD	49 ± 9
Median (IQR)	50 (40, 55)
Preference for a physician’s sex Male / Female / Neutral	24 /15 / 86
Preference for a physician’s age (< 40 years / 40–60 years / > 60 years / Neutral)	9 / 88 / 3 / 25
Number of consultations per year (≤1 / > 1)	48 / 77

Smile importance scored 8.5/10. There was no significant difference between men and women (*p* = 0.66) or between two groups of age < 48 y and ≥ 48 y (p = 0.66).

### Classification of the 7 competencies from the most to the least important

When classifying the 7 competencies by order of priority (Table 2), the majority opted for the medical expertise as their first choice (74.4%) and less than the half (48.8%) put the communication as second. Then followed the health advocate role (41.6% put it third), the collaborator (36.3% put it fourth), the professional (35.2% put it fifth), the leader (52% put it sixth) and the scholar (52% put it seventh).

**Table 2:** Classification of competencies in order of priority as perceived by patients

		A	B	C	D	E	F	G
First choice	n	93	16	3	4	5	2	2
	%	74.4%	12.8%	2.4%	3.2%	4.0%	1.6%	1.6%
Second choice	n	19	61	12	6	22	2	3
	%	15.2%	48.8%	9.6%	4.8%	17.6%	1.6%	2.4%
Third choice	n	3	18	52	25	18	3	6
	%	2.4%	14.4%	41.6%	20.0%	14.4%	2.4%	4.8%
Fourth choice	n	2	14	12	45	25	13	13
	%	1.6%	11.3%	9.7%	36.3%	20.2%	10.5%	10.5%
Fifth choice	n	0	9	17	19	44	19	17
	%	.0%	7.2%	13.6%	15.2%	35.2%	15.2%	13.6%
Sixth choice	n	3	4	19	7	8	65	19
	%	2.4%	3.2%	15.2%	5.6%	6.4%	52.0%	15.2%
Seventh choice	n	5	3	10	18	3	21	65
	%	4.0%	2.4%	8.0%	14.4%	2.4%	16.8%	52.0%

A, Medical Expert; B, Communicator; C, Health Advocate; D, Collaborator; E, Professional; F, Leader; G, Scholar

### Prioritization of sub-elements within each of the 7 competencies

A. Medical expertise: 56.8% of patients set as a priority the sub-element of clinical competence and expertise of the physician (Additional file 3: Table S1).
B. Communication: 36% preferred a doctor who listens to the patient (Additional file 3: Table S2).
C. Health advocate: 76.8% put a high weight on the work done with patients rather than with community for prevention and awareness (Additional file 3: Table S3).
D. Collaborator: 47.2% highlighted the skills of a physician capable of transferring the patient to another specialist (Additional file 3: Table S4).
E. Professional: 56% mentioned mostly the ethics of the physicians (Additional file 3: Table S5).
F. Leader: 30.4% chose the management of resources (Additional file 3: Table S6).
G. Scholar: 44.8% emphasized the continuing education of physicians (Additional file 3: Table S7).

### Krippendorff’s coefficient for evaluation of the reliability of choices

Krippendorff’s coefficient was assessed by taking into account the units, observers and pairs (Table 3). The unit is the number of items analyzed. Unit = 7 corresponds to the 7 dimensions analyzed in the question that classifies the 7 competencies; when analyzing each dimension or competency alone, the unit is then the number of sub-elements inside each dimension. The observers are the subjects who answered the questionnaire, that is, 125 subjects. The pair is the count of judgments (answers) done by the observers on the units.

**Table 3:** Krippendorff’s coefficient for evaluation of the reliability of choices

	Alpha coefficient	LL95%CI	UL95%CI	Units	Observers	Pairs
7 dimensions	0.4473	0.3954	0.4946	7	125	54,126
Choice A	0.2312	0.1462	0.3195	5	125	38,750
Choice B	0.0673	−0.0312	0.1622	5	125	38,750
Choice C	0.2844	0.1036	0.4821	2	125	15,500
Choice D	0.0298	−0.1237	0.1988	3	125	23,250
Choice E	0.2848	0.1826	0.3782	4	125	31,000
Choice F	0.0065	−0.1118	0.1238	4	125	31,000
Choice G	0.0586	−0.0549	0.1597	4	125	31,000

Krippendorff’s alpha coefficient = 1 depicts high reliability and coefficient = 0.0000 a null reliability; LL95%CI, lower limit of the 95% confidence interval; UL95%CI, higher limit of the 95% confidence interval;

A, Medical Expert; B, Communicator; C, Health Advocate; D, Collaborator; E, Professional; F, Leader; G, Scholar

We found a moderate agreement for the total 7 dimensions (A to G), a weak agreement for A, C and E and a null agreement for B, D, F and G (Table 3).

### Open-ended questions: how do you define the “good doctor” and the “bad doctor”?

**Fig. 1:**
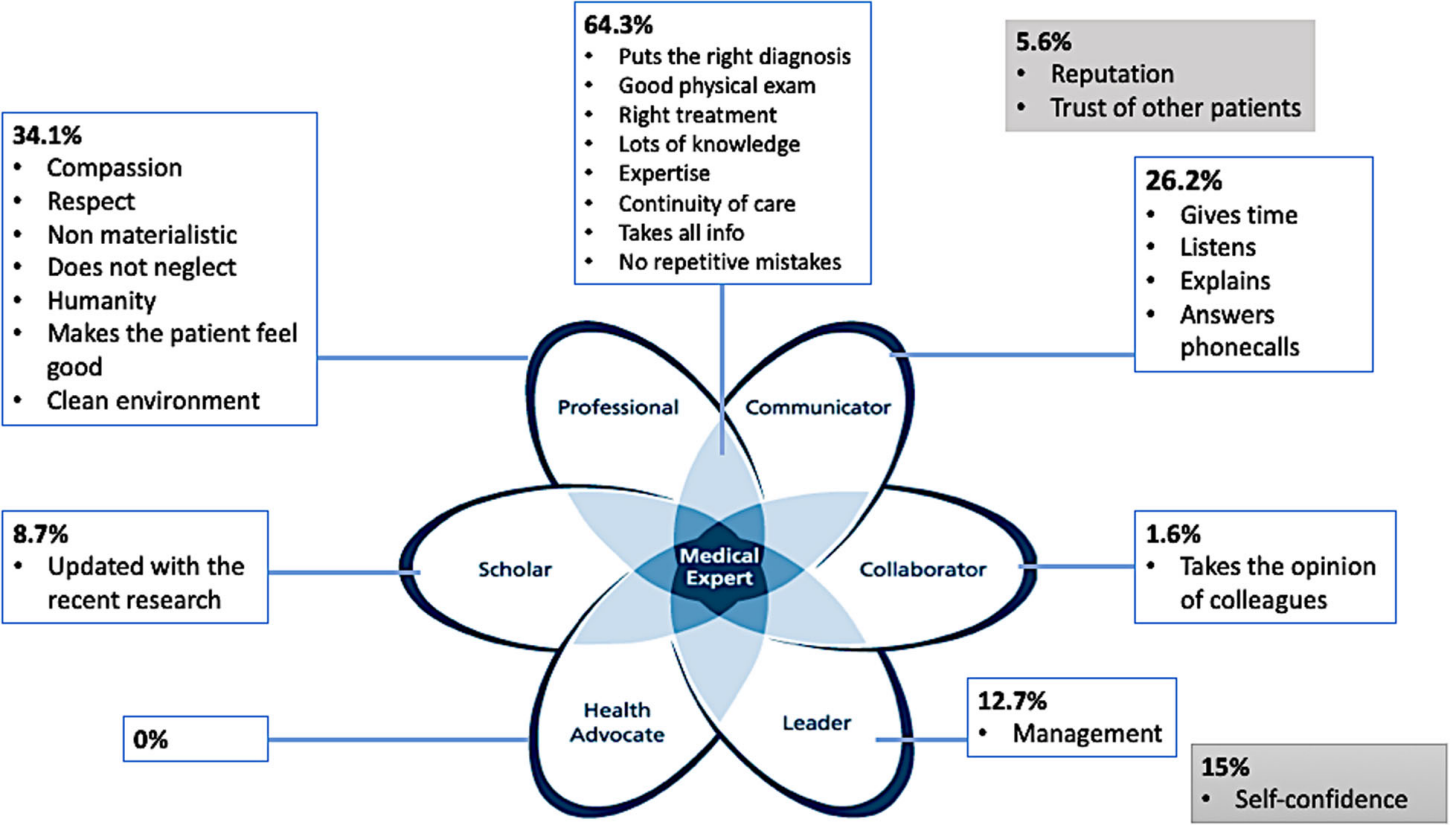
Definition of the “good doctor” by the 125 Lebanese patients: distribution of answers throughout the 7 competencies. Adapted from the CanMEDS 2015 flower diagram [7]

In the open-ended questions, patients defined the competent doctor in 185 answers and the non-competent one in 140 answers. A total of 325 answers were reported where three CanMEDS roles were mostly highlighted (Fig. 1): 64.3% of the patients mentioned features of the medical expert, 34.1% emphasized the high ethical attitude and 26.2% pointed out to communication. Just a small number of patients mentioned the scholar role and none the heath advocate.

In the group of patients who cited the medical expert characteristics, their mean age was 47.7 ± 17.6 years, 64.2% worked, 50.6% were males, 67.9% went to college, 39.5% defined a good or bad doctor based on “the right diagnosis” and 28.4% of them based on “the right treatment”.

In the group of patients who cited communication skills, their mean age was 46.2 ± 16.7 years, 75.8% worked, 57.6% were females, 78.8% went to college, 63.6% highlighted the physician’s listening skills and 48.5% wanted a physician who explains.

In the group of patients who cited the professionalism, their mean age was 47.8 ± 16.8 years, 67.4% worked, 53.5% were females, 74.4% went to college, 41.9% emphasized the doctor’s empathy, 30.2% feared neglect and lack of care, 23.3% claimed respect et 16% humanity.

In the group of patients who cited the high self-confident doctor (15% of patients), their mean age was 43.6 ± 17.3 years, 73.7% worked, 57.9% were females, 84.2% went to college, and no difference was depicted between men and women.

When mentioning the leadership skill, patients exclusively tackled the management of poor resources where patients see the good doctor as the one who provides the best quality of care with the minimum of tests (12.7%).

### Comparisons based on gender and age

Based on the open-ended questions, men slightly surpassed women in citing the role of medical expert (*p* = 0.043). No significant difference was noted in the age between those prioritizing a competency and those not.

## Discussion

This patient-centered study showed that Lebanese people seeking care favor the physician’s role of medical expert and put it at the top of the list of competencies. This finding was manifest in the closed and open-ended questions. It concurs well with the global perception of a competent doctor and reinforces the focus on this “sine qua non” competency in all schools of medicine [2, 21]. Medical expertise is placed at the heart of the CanMEDS 2015 flower diagram and all other competency frameworks agree that a good doctor should be excellent in questioning, examining, diagnosing and treating the patient [7, 15, 17]. In order to optimize the clinical expertise of medical graduates in Lebanon, some authors have suggested to start by reducing the variation in practices and proposed a unification of medical schools’ curricula [26]. Unexpectedly, our study found a higher proportion of men prioritizing the medical expert role despite the fact that the educational level across gender was similar. This finding needs to be assessed further in the future to confirm whether Lebanese women favor the interrelational competencies over the rational approach.

The second most preferred competency in the open-ended questions appears to be professionalism with the majority of answers putting stress on the physician’s ethical attitude. This is not surprising knowing that the roots of medicine are based on Hippocrates code of ethics [27, 28]. Professionalism has been one of the most rapidly integrated competencies in the Canadian program along with medical expertise and scholar roles [2]. However, it is still difficult to teach professionalism especially in the post-modern era where physicians carry a huge responsibility not only towards patients but also regarding their own well-being [29]. A lack of professionalism can be perceived very differently by regions and it may simply tackle the choice of words [30] or clothes [22]. Some authors have suggested three different ways to teach professionalism [31]. The most traditional physicians find the professionalism in values, humanitarian attitude, compassion, respect. A second group considers professionalism as a competency allowing physicians to show good behavior when performing a task at the bed of the patient. The third group integrates professionalism in a wider framework where a physician interacts within the community and aims for perfection in his practice [31]. Our patients have clearly expressed their need of the traditional human values in a physician and this is not surprising coming from an oriental background where religious values predominate whether Christians or Muslims.

Interestingly, the communicator role came out second in the patients’ classification of competencies and third in the open-ended questions. In most of the patients’ answers, « listening » was pointed out most among all the other sub-elements of communication. Listening to patients has been demonstrated as a crucial step in any health system that aims to achieve performance improvement and preserve the security of the patient by minimizing medical errors [32]. It is a quality that needs to be taught to medical students at their first clinical encounter with patients and it is essential in both oriental and occidental communities. Indeed, communication and professionalism have been picked up as most important by students in the Netherlands whereas active listening and verbal communication in critical clinical situations were also prioritized by their medical teachers [19, 20]. In 2017, in Poland, the « PRACTA study » developed an online program that teaches general practitioners the kind of words that old patients expect to hear when communicating their symptoms [33]. Finally, in our survey, as a part of both non verbal communication and compassion, the smile of a physician got a score of 8.5/10 stressing on the importance of a smile to reduce the psychological distance between individuals [34].

A remarkable finding in this study was the patient’s perception of just one aspect of leadership: the capacity of the physician to offer the best quality of care with a minimum of prescribed tests. This is a need that is aligned with the socioeconomic status of our developing country. Indeed, cost-effectiveness and efficiency are two characteristics necessary in a middle-income country. Managing and leading continuous improvement were not mentioned and this could be due to the resistance of the modern society to put doctors at the top of the hierarchy. This issue was largely discussed by Sonnenberg et al. that proposed to combine the leadership with the interprofessional collaboration and work seriously to teach this leadership’s team work to residents [35]. An educational program of collaborative leadership has been already implemented by the American College of Cardiology that believes that developing non-clinical skills of cardiology students can enhance the improvement of the cardiovascular health of patients [36, 37]. It would be interesting to integrate such program in the different specializations’ curricula of different schools of medicine in Lebanon and the world.

Of utmost importance is the lack of emphasis of patients on collaboration, health promotion and erudition mainly in the open-ended questions. In the closed questions, these three competencies came also after medical expertise and communication. Regarding health promotion, this competency appears to be neglected and underestimated by the Lebanese population and deserves to be discussed and promoted with the help of different stakeholders involved in the national health system. The erudite or scholar role was mentioned by a small number of patients who defined a good doctor as someone who performs research. This is a role that is usually more appreciated by physicians and students [2, 38] and it is not surprising that non-physicians neglect this aspect. However, erudition is an indirect factor that strengthens the medical expert role that was most chosen by our patients and deserves more attention. Although this study assesses the needs and perceptions of patients, one should remember that patients are not aware of all aspects that would improve a physician’s performance, especially when these physicians are simultaneously clinicians, teachers and researchers. Our results demonstrated the absence of patients’ perception of the importance of teaching. And this will enhance the challenge that physicians face to keep a balance between their clinical practice and their academic profession [39]. Unfortunately, without the academic aspect and its public recognition, medical profession cannot progress.

It is noteworthy that answers to open-ended questions included two ideas that are not mentioned in any of the CanMEDs competencies: the physician’s self-confidence and the notoriety. Self-confidence may be integrated with leadership or communication. Notoriety is the end-point of physicians’ performance and reflects the trust of patients. Effectively, all physicians aspire to get the trust of their community and several studies point out to the global crisis in the relationship patient-physician [40]. Trust, when achieved, can enhance patients’ compliance and intake of medication [41]. A group of family physicians evaluated 414 patients and found out that trust is dependent on ethics and communication, two competencies highlighted by our patients [42]. Besides self-confidence and notoriety, patients did not suggest other new concepts especially related to our post-modern society like the internet and advanced techonology. A study from the Netherlands surveying 102 gynecologists highlighted two new competencies to be addressed in the future, the entrepreneur and the technology user [43]. Another one evaluating 225 general practitioners showed that an online training was found to be more effective than reading a PDF text [31]. These two competencies need to be taken into consideration for the new generations.

In summary, the results of this study can help developing a curriculum that is competency-based in Lebanon and adapted to the context’s needs. Ultimately at the level of student’s evaluation, the Objective Structured Clinical Examination (OSCE), when well prepared, can identify all the non-clinical competencies of a medical student [44]. Indeed the virtual patient may help students acquire the communication and collaboration roles however the best way would be by encountering real patients and experiences [45]. In Lebanon, Yazigi et al. highlighted the « Role Model » of a physician, necessary to transfer all competencies to medical students [46]. These students will be themselves the example to be passed on to future generations and this can be seen as a dynamic heritage.

This study has some limitations. First an information bias is possible in the classification of competencies A to G in order of priority; many patients may have kept the order A to G by default. If these patients were interviewed, this bias would have been reduced. Anyways, the open-ended questions helped in clarifying the patients’ real priorities and were considered more accurate. The second limitation is that the questionnaire was piloted but not well validated. The third limitation is the high number of patients included from the Mount-Lebanon governorate and although it has the highest number of inhabitants compared to other governorates, 68% is overrepresenting this region. Despite these limitations, this study is the first to evaluate patients’ needs and perceptions before integrating a competency-based program in medical students training.

## Conclusions

This study revealed that Lebanese patients put the role of medical expert at the center of their expectations towards a competent doctor and this is in agreement with the global practice of medical schools. The six other non-clinical competencies of the CanMEDs framework are given different weights by patients with professionalism, communication and leadership emerging first. Therefore, Lebanese medical students should be trained early on these three competencies that can be integrated progressively in the pre- and post-graduate curriculum. Finally, patients’ underrating of the physician’s health advocate, teacher, researcher and scholar roles brings to light the necessity of educating the population on the importance of these key qualities for a “good doctor”.

## Supplementary information

**Additional file 1:** Lebanese version of the survey.

**Additional file 2:** English version of the Survey about the ideal physician.

**Additional file 3: Table S1.** Classification of the sub-elements of the Medical Expert. **Table S2.** Classification of the sub-elements of the Communicator. **Table S3.** Classification of the sub-elements of the Health Advocate. **Table S4.** Classification of the sub-elements of the Collaborator. **Table S5.** Classification of the sub-elements of the Professional. **Table S6.** Classification of the sub-elements of the Leader. **Table S7.** Classification of the sub-elements of the Erudite or Scholar.

## Data Availability

All data generated or analysed during this study are included in this published article [and its supplementary information files].

## Abbreviations

ACGME: Accreditation Council for Graduate Medical Education
CanMEDS: Canadian Medical Education Directions for Specialists
OSCE: Objective Structured Clinical Examination
