# Supplementary material for: How do Lebanese patients perceive the ideal doctor based on the CanMEDS competency framework?
Source: BMC Med Educ. 2019 Oct 29;19:399. doi: 10.1186/s12909-019-1837-y (PMC6821035; doi:10.1186/s12909-019-1837-y)
Supplement: Supplementary file 1 — Additional file 1: Lebanese version of the survey. [file 12909_2019_1837_MOESM1_ESM.pdf]

CODE

# دراسة حول الطبيب المثالي

هذه الدراسة تحتوي على أسئلة حول المواصفات المفضلة التي يتمنّى أن يراها المريض في طبيبه.

هذه الدراسة يقوم بها أطباء من جامعة القديس يوسف و هدفها تحسين الأداء الطبي.

نتمنى عليكم الاجابة على الاسئلة التالية دون ذكر الاسم. المشاركة بهذا الاستطلاع ليس وراءه استفادة مادية لأي طرف.

ولكم الشكر الجزيل

CODE

-١- سنة الولادة:

-٢- الجنس:

امرأة رجل

-٣- هل لديك شهادة جامعية؟

نعم كلا

-٤- العمل:

-٥- ما هو اختصاص الطبيب الذي يعالجك في أكثر الأوقات؟

-٦- هل هذا الطبيب امرأة أو رجل؟

امرأة رجل

-٧- ما هو العمر التقريبي لطبيبك؟

-٨- هل تفضّل أن يكون طبيبك إمرأة أم رجل؟

امرأة رجل لا يهم

-٩- في أي عمر تفضّل أن يكون طبيبك؟

تحت ٤٠ ٤٠ الى ٦٠ فوق ٦٠ لا يهم

-١٠- كم مرة تزور الطبيب سنويا؟

مرة أكثر من مرة

**-١١-** كيف تصف الطبيب المثالي، للإجابة على هذا السؤال الرجاء قراءة تفاصيل كل مقطع والإجابة على السؤال في آخره:

**-A- من لديه مهارة طبية Expert médical**

-1- من لديه معلومات واسعة في الطب واختصاصه و يؤمن لمريضه أعلى جودة في الرعاية الصحية

-2- من يأخذ جيدا كل المعلومات اللازمة من مريضه، من يفحص المريض بدقة، من يطلب الفحوصات المخبرية اللازمة، من يشخّص المرض جيدا و من يعرض للمريض الخطة لمعالجته حسب الأولوية في مشاكله الصحية

-3- من يعطي الدواء المناسب ويحرص أن يفسر للمريض عن المضاعفات

-4- من يحسن متابعة حالة مريضه

-5- من يحرص على سلامة مريضه

الرجاء ترتيب النقاط 1,2,3,4,5 في المقطع A من الاهم الى الاقل اهمية على السطر التالي————————

————————

**-B- من يحسن التواصل مع الاخرين Communicateur**

-1- من يعامل المريض وعائلته باحترام وتعاطف و من يتأهّل به عند دخوله العيادة ويحسن سياقة الحديث

-2- من يسمع للمريض دون أن يقاطعه لأخذ المعلومات المهمة و من يعطي وقتا طويلا للتحدث مع مريضه

-3- من يفسر جيدا للمريض المرض والعلاج

-4- من يشجع المريض وعائلته لطرح الأسئلة والاستفهام عن المرض والعلاج و من يحثّه الى المشاركة في قرارات تخص صحته

-5- من يدون كل المعلومات حول صحة المريض ويضعها تحت تصرفه عند الحاجة ويحافظ على السرية الطبية لملفاته

الرجاء ترتيب النقاط 1,2,3,4,5 في المقطع B من الاهم الى الاقل اهمية على السطر التالي————————

————————

CODE

**-C-من يشجع الوقاية الصحية Promoteur de la santé**

-1- من يعطي للمريض نصائح للوقاية من الأمراض

-2- من يكرّس وقتا لمحاضرات للتوعية عن الأمراض

الرجاء ترتيب النقاط 1,2 في المقطع C من الاهم الى الاقل اهمية على السطر التالي________________

__________

**-D- من يتعاون مع زملائه Collaborateur**

-1- من ينسّق مع زملائه من أجل صحة المريض

-2- من يتعامل مع زملائه باحترام و يتعامل جيدا مع اختلافات الرأي

-3- من يحول المريض الى اختصاصي اخر عند اللزوم و يتكلم معه او يكتب له رسالة

الرجاء ترتيب النقاط 1,2,3 في المقطع D من الاهم الى الاقل اهمية على السطر التالي______________

____________

**-E-من يحافظ على مهنية عالية Professionnel**

-1- من يتعامل مع المريض بمهنية وأخلاق طبية عالية

-2- من يحافظ على لياقة مهنة الطب حسب تطلبات مجتمعه

-3- من يحترم القوانين في مهنة الطب

-4- من يحافظ على رفاهته وصحته ليكون باحسن حال لتلبية حاجات المريض

الرجاء ترتيب النقاط 1,2,3,4 في المقطع E من الاهم الى الاقل اهمية على السطر التالي____________

______________

CODE

**F-من يحسن الادارة والقيادة Leader-**

1- من يطبق نظام التحسين الدائم للرعاية الصحية-

2- من يحسن اعطاء أفضل رعاية صحية ضمن الموارد المادية الضيقة-

3- من يجيد قيادة الموقف من أجل أفضل رعاية صحية-

4- من يدير جيدا مواعيده ووقته ويحسن ادارة جميع أعماله-

الرجاء ترتيب النقاط 1,2,3,4 في المقطع F من الاهم الى الاقل اهمية على السطر التالي————————————

**G-من يعلّم التلاميذ ويتعلّم دائما Erudit-**

1- من يقرأ دائما ويتبع خطة للتعلّم الدائم والمستمر-

2- من يعلّم التلاميذ مع الحرص على عدم تعريض المريض للأذى-

3- من يطبق دائما آخر المستجدات العلمية-

4- من يشجع الدراسات ويشارك في أبحاث علمية-

الرجاء ترتيب النقاط 1,2,3,4 في المقطع G من الاهم الى الاقل اهمية على السطر التالي————————————

**-١٢-** بعد قراءة عناوين المقاطع **A,B,C,D,E,F,G** الرجاء ترتيب النقاط **A,B,C,D,E,F,G** من الاهم الى الأقل أهمية

على السطر التالي————————————————————:

**-١٣-** ضع علامة من ١ الى ١٠ على أهمية ابتسامة الطبيب:

**-١٤-** بنظرك ما هي المواصفات و الصفة الأهم لتقول انّ الطبيب غير شاطر؟

**-١٥-** بنظرك ما هي المواصفات والصفة الاهم لتقول ان الطبيب شاطر؟
