# Supplementary material for: How do Lebanese patients perceive the ideal doctor based on the CanMEDS competency framework?
Source: BMC Med Educ. 2019 Oct 29;19:399. doi: 10.1186/s12909-019-1837-y (PMC6821035; doi:10.1186/s12909-019-1837-y)
Supplement: Supplementary file 2 — Additional file 2: English version of the Survey about the ideal physician. [file 12909_2019_1837_MOESM2_ESM.docx]

**Survey about the ideal physician**

This study includes questions about the qualities a patient would prefer to see in his physician.

This study is under the supervision of physicians from the Saint-Joseph University of Beirut and has the aim to improve the competencies of medical students and physicians.

We would appreciate your participation in this survey by replying to this questionnaire anonymously and we would like to inform you that there is no financial benefit from this study to anyone.

Thank you for your participation.

**Questionnaire**

1. Year of birth:
2. Sex: Female Male
3. Do you a college degree? Yes No
4. Work type:
5. Specialty of your physician:
6. Is your physician a woman or man? Woman Man
7. What is the approximate age of your physician?
8. Would you prefer a physician female or male? Woman Man It doesn’t matter
9. What is your preference for the physician’s age? <40 40-60 >60 It doesn’t matter
10. How frequently do you see your physician yearly? Once More than once
11. How do you describe the ideal physician, in order to answer to this question please read the details of each of the following paragraphs and answer at the end of each paragraph:
12. **The Medical Expert** **is the one who:**

1. Has solid knowledge and applies it to offer the best care

2. Is able to get a good medical history, perform a rigorous physical exam, asks for the necessary paraclinical tests, puts the right diagnosis and proposes to the patient a clear management of his illness based on priorities

3. Prescribes the appropriate treatment and explains about its side-effects

4. Ensures a continuity in the care and treatment of the patient

5. Preserves the patient’s safety

Please put in order from the most important to the least important the points 1, 2, 3, 4, 5 from paragraph A: __________________________

1. **The Communicator is**:

1. Communicates with the patient and the family with respect and compassion and leads a good conversation

2. Listens to the patient without interrupting and gives the necessary time to get the important informations

3. Explains to the patient the disease and treatment

4. Encourages the patient and the family to ask questions to understand more the disease and take part in the decisions

5. Documents all informations while preserving confidentiality

Please put in order from the most important to the least important the points 1, 2, 3, 4, 5 from paragraph B: __________________________

1. **The Health Advocate is the one who:**

1. Works at the level of patients to ensure the prevention and awareness of diseases

2. Works at the level of the community to ensure the prevention and awareness of diseases

Please put in order from the most important to the least important the points 1, 2 from paragraph C: _____________________________

1. **The Collaborator is the one who:**

1. Collaborates effectively with other healthcare professionals

2. Knows how to deal with conflicts and misunderstandings with colleagues

3. Is capable of transferring the care of the patient to another colleague if necessary

Please put in order from the most important to the least important the points 1, 2, 3 from paragraph D: ______________________________

1. **The professional is:**

1. Acts with his patients with high ethics

2. Acts in response to the society’s expectation of professionalism

3. Follows the laws of the medical profession

4. Preserves his well-being in order to give the best care to patients

Please put in order from the most important to the least important the points 1, 2, 3, 4 from paragraph E: ___________________________________

1. **The Leader is:**

1. Applies a policy of improvement in his care for patients

2. Ensures best quality with minimal use of resources

3. Leads well to ensure best quality of care

4. Manages well his time and work

Please put in order from the most important to the least important the points 1, 2, 3, 4 from paragraph F: ___________________________________

1. **The Erudite is:**

1. Reads regularly and follows a plan for continuous education

2. Teaches students without jeopardizing the patient’s safety

3. Is up to date in his medical knowledge

4. Is involved in research

Please put in order from the most important to the least important the points 1, 2, 3, 4 from paragraph G: _____________________________________

1. **After reading all the paragraphs from A to G, please put in order the points A, B, C, D, E , F , G, from the most important to the least important for you: ____________________________**
2. Put a grade from 1 to 10 to the physician’s smile impact:
3. From your point of view, what are the qualifications you find important to say the physician is not a good doctor?
4. From your point of view, what are the qualifications you find important to say the physician is a good doctor?
