# Supplementary material for: How do Lebanese patients perceive the ideal doctor based on the CanMEDS competency framework?
Source: BMC Med Educ. 2019 Oct 29;19:399. doi: 10.1186/s12909-019-1837-y (PMC6821035; doi:10.1186/s12909-019-1837-y)
Supplement: Supplementary file 3 — Additional file 3: Table S1. Classification of the sub-elements of the Medical Expert. Table S2. Classification of the sub-elements of the Communicator. Table S3. Classification of the sub-elements of the Health Advocate. Table S4. Classification of the sub-elements of the Collaborator. Table S5. Classification of the sub-elements of the Professional. Table S6. Classification of the sub-elements of the Leader. Table S7. Classification of the sub-elements of the Erudite or Scholar. [file 12909_2019_1837_MOESM3_ESM.docx]

**Table S1. Classification of the sub-elements of the Medical Expert**

|  | **Answer 1** | | **Answer 2** | | **Answer 3** | | **Answer 4** | | **Answer 5** | |
| --- | --- | --- | --- | --- | --- | --- | --- | --- | --- | --- |
|  | n | % | n | % | n | % | n | % | n | % |
| **First choice** | 30 | 24.0% | 71 | **56.8%** | 4 | 3.2% | 5 | 4.0% | 15 | 12.0% |
| **Second choice** | 46 | **36.8%** | 32 | 25.6% | 24 | 19.2% | 18 | 14.4% | 5 | 4.0% |
| **Third choice** | 17 | 13.6% | 12 | 9.6% | 59 | **47.2%** | 27 | 21.6% | 10 | 8.0% |
| **Fourth choice** | 13 | 10.4% | 3 | 2.4% | 24 | 19.2% | 56 | **44.8%** | 29 | 23.2% |
| **Fifth choice** | 19 | 15.2% | 7 | 5.6% | 14 | 11.2% | 19 | 15.2% | 66 | **52.8%** |

**Answer 1** : The one who has solid knowledge and applies it to offer the best care; **Answer** 2 : The one who is able to get a good medical history, perform a rigorous physical exam, asks for the necessary paraclinical tests, puts the right diagnosis and proposes to the patient a clear management of his illness based on priorities; **Answer** 3 : The one who prescribes the appropriate treatment and explains about its side-effects; **Answer** 4 : The one who ensures a continuity in the care and treatment of the patient; **Answer** 5 : The one who preserves the patient’s safety

**Table S2. Classification of the sub-elements of the Communicator**

|  | **Answer 1** | | **Answer 2** | | **Answer 3** | | **Answer 4** | | **Answer 5** | |
| --- | --- | --- | --- | --- | --- | --- | --- | --- | --- | --- |
|  | n | % | n | % | n | % | n | % | n | % |
| **First choice** | 37 | 29.6% | 45 | **36.0%** | 26 | 20.8% | 7 | 5.6% | 10 | 8.0% |
| **Second choice** | 12 | 9.6% | 38 | **30.4%** | 34 | 27.2% | 19 | 15.2% | 22 | 17.6% |
| **Third choice** | 20 | 16.0% | 15 | 12.0% | 39 | 31.2% | 24 | 19.2% | 27 | **21.6%** |
| **Fourth choice** | 17 | 13.6% | 20 | 16.0% | 21 | 16.8% | 50 | **40.0%** | 17 | 13.6% |
| **Fifth choice** | 38 | 30.4% | 8 | 6.4% | 5 | 4.0% | 25 | 20.0% | 49 | **39.2%** |

**Answer** 1 : Communicates with the patient and the family with respect and compassion and leads a good conversation; **Answer** 2 : Listens to the patient without interrupting and gives the necessary time to get the important informations ; **Answer** 3 : Explains to the patient the disease and treatment; **Answer** 4 : Encourages the patient and the family to ask questions to understand more the disease and take part in the decisions; **Answer** 5 : Documents all informations while preserving confidentiality

**Table S3. Classification of the sub-elements of the Health Advocate**

|  | **Answer 1** | | **Answer 2** | |
| --- | --- | --- | --- | --- |
|  | n | % | n | % |
| **First choice** | 96 | **76.8%** | 29 | 23.2% |
| **Second choice** | 29 | 23.2% | 96 | **76.8%** |

**Answer** 1 : Work at the level of patients to ensure the prevention and awareness of diseases; **Answer** 2 : Work at the level of the community to ensure the prevention and awareness of diseases

**Table S4. Classification of the sub-elements of the Collaborator**

|  | **Answer 1** | | **Answer 2** | | **Answer 3** | |
| --- | --- | --- | --- | --- | --- | --- |
|  | n | % | n | % | n | % |
| **First choice** | 45 | 36.0% | 21 | 16.8% | 59 | **47.2%** |
| **Second choice** | 58 | **46.4%** | 36 | 28.8% | 31 | 24.8% |
| **Third choice** | 22 | 17.6% | 68 | **54.4%** | 35 | 28.0% |

**Answer** 1 : Collaborates effectively with other healthcare professionals; **Answer** 2 : Knows how to deal with conflicts and misunderstandings with colleagues; **Answer** 3 : Capable of transferring the care of the patient to another colleague if necessary

**Table S5. Classification of the sub-elements of the Professional**

|  | **Answer 1** | | **Answer 2** | | **Answer 3** | | **Answer 4** | |
| --- | --- | --- | --- | --- | --- | --- | --- | --- |
|  | n | % | n | % | n | % | n | % |
| **First choice** | 70 | **56.0%** | 21 | 16.8% | 22 | 17.6% | 12 | 9.6% |
| **Second choice** | 24 | 19.2% | 42 | 33.6% | 47 | **37.6%** | 12 | 9.6% |
| **Third choice** | 19 | 15.2% | 54 | **43.2%** | 41 | 32.8% | 11 | 8.8% |
| **Fourth choice** | 12 | 9.6% | 8 | 6.4% | 15 | 12.0% | 90 | **72.0%** |

**Answer** 1 : Acts with his patients with high ethics; **Answer** 2 : Acts in response to the society’s expectation of professionalism; **Answer** 3 : Follows the laws of the medical profession ; **Answer** 4 : Preserves his well-being in order to give the best care to patients

**Table S6. Classification of the sub-elements of the Leader**

|  | **Answer 1** | | **Answer 2** | | **Answer 3** | | **Answer 4** | |
| --- | --- | --- | --- | --- | --- | --- | --- | --- |
|  | n | % | n | % | n | % | n | % |
| **First choice** | 27 | 21.6% | 26 | 20.8% | 38 | **30.4%** | 34 | 27.2% |
| **Second choice** | 30 | 24.0% | 41 | **32.8%** | 29 | 23.2% | 25 | 20.0% |
| **Third choice** | 34 | 27.2% | 32 | 25.6% | 39 | **31.2%** | 20 | 16.0% |
| **Fourth choice** | 34 | 27.2% | 26 | 20.8% | 19 | 15.2% | 46 | **36.8%** |

**Answer** 1 : Applies a policy of improvement in his care for patients; **Answer** 2 : Ensures best quality with minimal use of resources; **Answer** 3 : Leads well to ensure best quality of care; **Answer** 4 : Manages well his time and work

**Table S7. Classification of the sub-elements of the Erudite or Scholar**

|  | **Answer 1** | | **Answer 2** | | **Answer 3** | | **Answer 4** | |
| --- | --- | --- | --- | --- | --- | --- | --- | --- |
|  | n | % | n | % | n | % | n | % |
| **First choice** | 56 | **44.8%** | 26 | 20.8% | 26 | 20.8% | 17 | 13.6% |
| **Second choice** | 25 | 20.0% | 32 | 25.6% | 27 | 21.6% | 41 | **32.8%** |
| **Third choice** | 29 | 23.2% | 23 | 18.4% | 51 | **40.8%** | 22 | 17.6% |
| **Fourth choice** | 15 | 12.0% | 44 | 35.2% | 21 | 16.8% | 45 | **36.0%** |

**Answer** 1 : Reads regularly and follows a plan for continuous education; **Answer** 2 : Teaches students without jeopardizing the patient’s safety; **Answer** 3 :Is up to date in his medical knowledge; **Answer** 4 : Is involved in research
